# Supplementary material for: Civil society perspectives on tuberculosis care for people living with HIV in Brazil: A study informed by Social Representations Theory
Source: PLOS Glob Public Health. 2026 Mar 18;6(3):e0006119. doi: 10.1371/journal.pgph.0006119 (PMC12998840; doi:10.1371/journal.pgph.0006119)
Supplement: S1 Table — (DOCX) [file pgph.0006119.s001.docx]

**S1 Table. COREQ (COnsolidated criteria for REporting Qualitative research) Checklist**

| **Item No.** | **Topic** | **Guide Question/Description** | September 23, 2025. |
| --- | --- | --- | --- |
| **Domain 1:** Research team and reflexivity | | | |
| 1 | Interviewer/facilitator | Which author/s conducted the interview or focus group? | Self-administered |
| 2 | Credentials | What were the researcher’s credentials? E.g. PhD, MD | Yes. The researchers were nurses, including one doctoral student and four master’s students. There were also four undergraduate nursing students in their fourth year of study. |
| 3 | Occupation | What was their occupation at the time of the study? | Yes. The researchers were students, including one doctoral student and four master’s students, affiliated with a Graduate Program in Nursing, and four undergraduate students, affiliated with an Undergraduate Program in Nursing. |
| 4 | Gender | Was the researcher male or female? | Yes. The study was composed of five female researchers and four male researchers. |
| 5 | Experience and training | What experience or training did the researcher have? | The researchers had a background in nursing and prior experience in qualitative research, particularly in areas related to public health, such as HIV/AIDS and tuberculosis surveillance. |
| 6 | Relationship established | Was a relationship established prior to study commencement? | Yes. Prior contact was made with the mediators (representatives of civil society). |
| 7 | Participant knowledge of the interviewer | What did the participants know about the researcher? e.g. personal goals, reasons for doing the research | The purpose of the research was explained to the mediators, as well as that the study was part of a research group linked to a Public University in Northwest Paraná. |
| 8 | Interviewer characteristics | What characteristics were reported about the inter viewer/facilitator? e.g. Bias, assumptions, reasons and interests in the research topic | The facilitators were representatives of civil society, previously trained to conduct interviews, who were interested in the topic. |
| **Domain 2:** Study design | | | |
| 9 | Methodological orientation and Theory | What methodological orientation was stated to underpin the study? e.g. grounded theory, discourse analysis, ethnography, phenomenology, content analysis | Theory of Social Representations. |
| 10 | Sampling | How were participants selected? e.g. purposive, convenience, consecutive, snowball | Convenience and intentionality. |
| 11 | Method of approach | How were participants approached? e.g. face-to-face, telephone, mail, email | The facilitators were approached through prior contact via email, where the purpose of the research was explained and subsequently the facilitators themselves made contact with the other representatives of civil society. |
| 12 | Sample size | How many participants were in the study? | 37 participants representing society were included. |
| 13 | Non-participation | How many people refused to participate or dropped out? Reasons? | N/A. |
| 14 | Setting of data collection | Where was the data collected? e.g. home, clinic, workplace | An accessible and private institutional location. |
| 15 | Presence of nonparticipants | Was anyone else present besides the participants and researchers? | N/A. |
| 16 | Description of sample | What are the important characteristics of the sample? e.g. demographic data, date | The study sample comprised 37 participants, all representing civil society. Interviews were conducted between January and July 2025 across five Brazilian state capitals—Porto Alegre, Manaus, Recife, Campo Grande, and Rio de Janeiro—selected due to their significance in the national landscape of social control over HIV and tuberculosis. |
| 17 | Interview guide | Were questions, prompts, guides provided by the authors? Was it pilot tested? | The authors developed a questionnaire with open-ended questions, which served as a guide for mediators (civil society representatives) in conducting the focus groups. |
| 18 | Repeat interviews | Were repeat inter views carried out? If yes, how many? | Only one interview was conducted with each focus group. |
| 19 | Audio/visual recording | Did the research use audio or visual recording to collect the data? | Audio recording. |
| 20 | Field notes | Were field notes made during and/or after the inter view or focus group? | N/A. |
| 21 | Duration | What was the duration of the inter views or focus group? | Average duration of 60 minutes. |
| 22 | Data saturation | Was data saturation discussed? | Yes. “Data collection was terminated when the speeches began to present repetitive content and a lack of new relevant information.” |
| 23 | Transcripts returned | Were transcripts returned to participants for comment and/or | N/A. |
| **Domain 3:** analysis and findings | | | |
| 24 | Number of data coders | How many data coders coded the data? | Nine researchers |
| 25 | Description of the coding tree | Did authors provide a description of the coding tree? | Yes. The data was organized into three main categories. |
| 26 | Derivation of themes | Were themes identified in advance or derived from the data? | Derived from the data. |
| 27 | Software | What software, if applicable, was used to manage the data? | N/A. |
| 28 | Participant checking | Did participants provide feedback on the findings? | N/A. |
| 29 | Quotations presented | Were participant quotations presented to illustrate the themes/findings? Was each quotation identified? e.g. participant number | Yes. The citations were presented using the names of the capitals. |
| 30 | Data and findings consistent | Was there consistency between the data presented and the findings? | Yes. |
| 31 | Clarity of major themes | Were major themes clearly presented in the findings? | Yes. They were clearly presented, described and organized into categories, according to the theoretical framework established by the researchers. |
| 32 | Clarity of minor themes | Is there a description of diverse cases or discussion of minor themes? | Yes. In addition to the categories listed, the study enabled the identification and description of different social contexts among Brazilian capitals. |
