# Supplementary material for: Civil society perspectives on tuberculosis care for people living with HIV in Brazil: A study informed by Social Representations Theory
Source: PLOS Glob Public Health. 2026 Mar 18;6(3):e0006119. doi: 10.1371/journal.pgph.0006119 (PMC12998840; doi:10.1371/journal.pgph.0006119)
Supplement: S2 Table — (DOCX) [file pgph.0006119.s002.docx]

| **City (Region)** | **TB incidence**  **(per 100k hab., 2024)** | **TB-HIV coinfection (%)** | **n participants** | **Gender (F/M)** | **Age range (years)** |
| --- | --- | --- | --- | --- | --- |
| Manaus (North) | 127,3 | 81,3 | 7 | 4F/3M | (22 - 39) |
| Recife (Northeast) | 97,6 | 81,7 | 9 | 3F/3M | (47 - 64) |
| Campo Grande (Central-West) | 53 | 93,1 | 7 | 5F/2M | (37-51) |
| Rio de Janeiro (Southeast) | 96,1 | 91,0 | 6 | 5F/1M | (32 - 49) |
| Porto Alegre (South) | 75,4 | 90,4 | 6 | 4F/2H | (33 - 50) |

S2 Table. Epidemiological indicators of study sites and participant characteristics by city.
