# Supplementary material for: Civil society perspectives on tuberculosis care for people living with HIV in Brazil: A study informed by Social Representations Theory
Source: PLOS Glob Public Health. 2026 Mar 18;6(3):e0006119. doi: 10.1371/journal.pgph.0006119 (PMC12998840; doi:10.1371/journal.pgph.0006119)
Supplement: S1 Text — (DOCX) [file pgph.0006119.s003.docx]

**S1 Text. Focus group semi-structured interview guide**

**Welcome message**

Welcome! We sincerely thank you for participating in this focus group, which aims to better understand civil society’s perceptions regarding the management of tuberculosis among people living with HIV (PLHIV). We would like to hear about your experiences, challenges, and suggestions to improve these actions in partnership with health services.

**Instructions** To ensure a productive and respectful discussion, please consider the following guidelines:

- **Active participation:** Everyone is invited to share experiences and perspectives. There are no right or wrong answers.
- **Respect and active listening:** Please respect others’ contributions, avoid interruptions, and foster a welcoming dialogue.
- **Confidentiality:** To ensure a safe space, we ask that information shared here remains within the group and is not disclosed externally.
- **Focus on the topic:** Our time is limited, so we kindly ask that contributions remain concise and centered on the proposed themes.
- **Recording of discussions:** The session will be audio-recorded for subsequent analysis. Data will be used strictly for research purposes and discarded after completion. No names or personal identifiers will be directly linked to responses.

**Interview guide**

| **Theme** | **Guiding Question** |
| --- | --- |
| **Perceptions of TB among PLHIV** | 1. How do you perceive tuberculosis among people living with HIV in your community? |
| **Knowledge and awareness** | 2. Do you think people living with HIV are aware of the risks of tuberculosis and the importance of screening? Why? |
| **Access to diagnosis** | 3. Have you ever witnessed a situation in which a person living with HIV had difficulties accessing tuberculosis diagnostic tests? What happened? |
| **Preventive treatment (TPT)** | 4. In your experience, is tuberculosis preventive treatment (TPT) offered and accepted by people living with HIV? What are the main challenges? |
| **Barriers to treatment** | 5. What do you perceive as the main barriers for people living with HIV to initiate and complete TPT? |
| **Civil society interaction** | 6. How would you describe the relationship between your NGO/organization and health services regarding tuberculosis care among people living with HIV? |
| **Health services openness** | 7. Do you feel that health services are open to listening and working in partnership with civil society in the fight against tuberculosis? |
| **Strategies to improve access** | 8. In your opinion, what could be done to increase tuberculosis screening and preventive treatment among people living with HIV? |
| **Best practices and initiatives** | 9. Have you participated in or are you aware of any initiatives that helped expand access to tuberculosis screening and preventive treatment? |
| **Final considerations** | 10. Is there anything else about tuberculosis and HIV that you would like to share? |
