# Supplementary material for: Civil society perspectives on tuberculosis care for people living with HIV in Brazil: A study informed by Social Representations Theory
Source: PLOS Glob Public Health. 2026 Mar 18;6(3):e0006119. doi: 10.1371/journal.pgph.0006119 (PMC12998840; doi:10.1371/journal.pgph.0006119)
Supplement: S3 Table — (DOCX) [file pgph.0006119.s004.docx]

S3 Table. Saturation matrix of thematic codes across focus group discussions

| **Focus Group** | **City (Region)** | **Date of interview** | **Participants** | **No. of codes identified** | **No. of new codes** | **Cumulative total** | **% of new codes** |
| --- | --- | --- | --- | --- | --- | --- | --- |
| FG1 | Manaus (North) | 21/07/2025 | 7 | 18 | 18 | 18 | 100% |
| FG2 | Recife (Northeast) | 17/03/2025 | 9 | 9 | 4 | 22 | 18% |
| FG3 | Rio de Janeiro (Southeast) | 11/04/2025 | 6 | 12 | 3 | 25 | 12% |
| FG4 | Campo Grande (Midwest) | 30/04/2025 | 9 | 16 | 5 | 30 | 17% |
| FG5 | Porto Alegre (South) | 21/07/2025 | 6 | 9 | 3 | 33 | 9% |
