# Supplementary material for: Civil society perspectives on tuberculosis care for people living with HIV in Brazil: A study informed by Social Representations Theory
Source: PLOS Glob Public Health. 2026 Mar 18;6(3):e0006119. doi: 10.1371/journal.pgph.0006119 (PMC12998840; doi:10.1371/journal.pgph.0006119)
Supplement: S4 Table — (DOCX) [file pgph.0006119.s005.docx]

**S4 Table. Codebook of thematic categories and subcategories**

| **Category** | **Subcategory** | **Description** | **Illustrative Quote** |
| --- | --- | --- | --- |
| **1. Social and institutional neglect of tuberculosis** | **Environmental conditions** | Geographic isolation, drought, and lack of local health infrastructure hinder access to timely diagnosis and treatment. | “The medication is not available immediately. They told him to come back in three months to see if he could get an appointment in pulmonology to obtain the medication.” (P2, Recife) |
|  | **Socioeconomic barriers (food insecurity, prisons, poverty)** | Patients are unable to adhere to treatment in contexts of hunger or degrading living conditions. | “I think this aspect of public policy needs to be implemented. If a person has adequate nutrition and a suitable living space […] it’s useless to give a lot of medication to someone who doesn’t have the slightest chance of survival.” (P2, Campo Grande) |
|  | **Lack of basic infrastructure** | Absence of essential diagnostic tools such as X-ray machines in primary health services. | “In the countryside there is not even an X-ray.” (P2, Manaus) |
|  | **State omission** | Lack of governmental response despite the availability of epidemiological data. | “People think tuberculosis no longer exists.” (P3, Porto Alegre) |
|  | **Service deterioration** | Perceived worsening of service provision compared to previous years. | “What is lacking is for the Family Health Program to enter the territory and carry out prevention and health promotion work. Over time, promotion and prevention have been declining.” (P1, Rio de Janeiro) |
|  | **Unavailability of medications in health services** | Reports of shortages of TB and HIV drugs. | “Do you know what medication is given inside [the prisons] for tuberculosis? Dipyrone.” (P6, Campo Grande) |
|  | **Absence of campaigns (restricted to symbolic dates)** | Information limited to commemorative occasions. | “There is a lack of television campaigns. We used to see campaigns; you know? [...]” (P6, Rio de Janeiro) |
|  | **Right to information denied** | Users not receiving basic guidance in health services. | “There is a lack of information for people [living with HIV] to become aware of their situation and the risks they are facing.” (P2, Recife) |
|  | **Programmatic fragility** | Preventive therapy or protocols implemented only in external projects, not as standard care. | “From my observation, from my experience, when a person manages to see a doctor, the last thing, the last hypothesis considered, is tuberculosis.” (P2, Campo Grande) |
|  | **Need for professional sensitization** | Lack of empathy and communication, no structures for integrated psychosocial support | “Community health agents lack the sensitivity to convey this information and provide guidance; they do not have the proper tact to deal with certain populations.” (P3, Campo Grande) |
| **2. Stigma, poverty, and social exclusion in the context of coinfection** | **TB as stigma** | Social judgments and shame associated with TB diagnosis | “If I have tuberculosis, people will speak badly of me because I had tuberculosis.” (P1, Porto Alegre) |
|  | **Double burden of coinfection** | Coinfection with HIV intensifying psychological suffering and discrimination | “We who live with HIV are already very vulnerable, and when we arrive at the health service and encounter that barrier, everything collapses. We even lose the motivation to continue. Now imagine me being told one more thing, that it’s TB.” (P3, Recife) |
|  | **Perceived fatality** | TB described as a “death sentence” or fatal threat among PLHIV | “Tuberculosis is a breath toward death for those with HIV.” (P5, Rio de Janeiro) |
|  | **Discrimination in health services** | Experiences of neglect, distancing, and prejudice from health professionals | “A patient said they would pick up the medication, and the nurse handed it through the window because she didn’t want to have contact with a patient with tuberculosis.” (P4, Manaus) |
|  | **Socioeconomic barriers** | Poverty, hunger, and lack of adequate housing hindering adherence to treatment | “TB is still a disease affecting poor people, due to issues like basic sanitation and social benefits… TB occurs precisely among those experiencing social vulnerability.” (P4, Recife) |
|  | **Territorial vulnerability** | Exclusion in favelas, slums, and prisons limiting prevention and care | “The greatest barrier here in the Complexo do Alemão is this commitment to the territory.” (P7, Rio de Janeiro) |
|  | **Treatment abandonment** | Interruption of TB and HIV treatment due to vulnerability and lack of support. | “Once diagnosed with tuberculosis, she becomes isolated from others.” (P2, Campo Grande) |
| **3. Civil society and NGOs as mediators of care** | **NGOs bridging populations and services** | NGOs connecting vulnerable groups to formal health services | “We face, within the favela territory, a very serious issue: invisible people who do not attend health units. They simply don’t go. So, as an institution or non-governmental organization operating in the territory, we have this role.” (P1, Rio de Janeiro) |
|  | **Accessible information** | Civil society disseminating knowledge in everyday, non-technical language | “That is why the community network operates with people from the territory speaking to one another. […] You build your own language without using the medical, technical, or entirely formal language.” (P1, Rio de Janeiro) |
|  | **Rights advocacy** | Organizations defending health as a constitutional right, including for incarcerated populations | “We need to look and see, and perhaps determine what can be done, advocating for this focused attention. They are deprived of liberty, having lost it for a time, but they have not lost their rights, including the right to health, which is guaranteed by the Constitution.” (P5, Campo Grande) |
|  | **Emotional and community support** | NGOs as safe spaces of trust, attentive listening, and mutual care | “We care a lot for each other, we observe each other closely, we learn a great deal from one another, and we greatly support one another.” (P6, Recife) |
|  | **Challenges to institutional integration** | Reports of insufficient recognition, lack of funding, and exclusion from decision-making | “Healthcare professionals do not recognize civil society as partners.” (P1, Recife) |
|  | **Political and historical role** | Civil society as co-producer of care and as a driving force in national HIV/TB policy achievements | “Civil society was the driving force that positioned Brazil as a global reference in HIV-AIDS treatment. Thus, organized civil society, when taking to the streets and addressing stigma and LGBTQIA+ groups […] demonstrates in Brazil that public policy truly benefits from the contribution of a partnership with organized civil society.” (P1, Campo Grande) |
