# Supplementary material for: Civil society perspectives on tuberculosis care for people living with HIV in Brazil: A study informed by Social Representations Theory
Source: PLOS Glob Public Health. 2026 Mar 18;6(3):e0006119. doi: 10.1371/journal.pgph.0006119 (PMC12998840; doi:10.1371/journal.pgph.0006119)
Supplement: Attachment — Submitted filename: PLOS_GPH_Review_Detailed.pdf [file pgph.0006119.s006.pdf]

Title: Civil society view of TB care for PWHA as service users in Brazil: a study informed by Social Representations Theory

Overall Recommendation: Minor Revision

This review is in line with the PLOS Global Public Health reviewer guidelines, looking at validity, methodological soundness, clarity, and strength of contribution to the discipline.

Main Claims and Significance

The manuscript argues the view from civil society provides us with new insights into how the TB–HIV coinfection is constructed through stigma, institutional abdication, territorial inequity, and the mediating role of NGOs in care. These claims mean a lot to the world's public health, particularly with respect to human rights, community involvement, and equity in TB–HIV control. The manuscript presents an innovative lens by applying Social Representations Theory to study civil society actors—a previously neglected group in TB–HIV literature.

Placement in the Literature

The authors clearly situate the study within the current research on TB-HIV, stigma, and social determinants. They make references to international mega-facilities and national epidemiological databases. The literature review is comprehensive, current, and appropriately scoped. A clear lacuna in the civil society perspective is highlighted by the manuscript and discussion as relevant.

Data and Analyses Supporting Claims

The material thoroughly supports the arguments made about the theme. The analysis is thorough and based on inductive and SRT-informed deductive coding, triangulation, and saturation control. The participant quotes are well-chosen and effectively demonstrate the central themes. No additional data appears necessary. The conclusions are sound and not overstated.

Protocol and Deviations

Since this is a qualitative study, no formal assessment of protocol deviation will be required. The approaches followed recognized qualitative criteria, such as COREQ, ethical consent, and clear documentation on how the data were analyzed.

Protocol Availability or Supplemental Methods

While not a requirement, authors might be interested in submitting their full interview guide (which I assume is already supplementary) and perhaps the final codebook as supporting information. Those additions would improve the transparency of the course, although the analysis is already thorough enough for most purposes without them.

Suitability for Publication or Resubmission

The manuscript is acceptable for publication with minor revisions. It demonstrates clear potential, methodological rigor, and public health relevance. The relevant improvements are technical and mostly for the sake of clearness, to avoid repetition, or to enhance understanding, but not so much about actually introducing more valid knowledge as such.

Data Availability Requirements

In the case of qualitative research with sensitive human data, this restricted access, as detailed in our Data Availability Statement, is acceptable and in accordance with PLOS policy. The data supporting the study's findings are available from the corresponding authors upon reasonable request, following ethical approval.

Reporting Guidelines Compliance

The manuscript adheres to COREQ and general qualitative reporting standards. There are no other guidelines (CONSORT, STROBE, MIAME) that apply. Ethical approvals and participant protection are well documented.

Methodological Detail and Reproducibility

The methods are presented with enough detail to enable the analysis to be reproduced. Explanations of the recruitment, facilitation, transcription, analytic coding, and saturation monitoring are consistently clear.

Software Availability

No code that has a public release requirement was generated for the purpose of this study and is therefore not applicable.

Organization and Writing Quality

The article is well-written and, in general, easy to follow. Nonetheless, there is some degree of redundancy in the Discussion and the Conclusion that can be reduced. The abstract is too densely written and should be rewritten to make it clearer and more concise. A little linguistic polishing would help to make it more readable to non-specialists.

Recommendations to Strengthen the Paper

Condense the abstract to better illuminate what is new.

Eliminate repetition in the Discussion and Conclusion.

Create standardized definitions around “neglect,” “erasure,” and “invisibility.”

Explain why this study adds something new: civil society as co-producers of care.

Break up long paragraphs for easier reading.

Conclusion

This is a well-written and relevant paper that meets the publication criteria of PLOS Global Public Health. The article will contribute important new information to the existing literature, with some modifications made for clarity and brevity.
