# Supplementary material for: Civil society perspectives on tuberculosis care for people living with HIV in Brazil: A study informed by Social Representations Theory
Source: PLOS Glob Public Health. 2026 Mar 18;6(3):e0006119. doi: 10.1371/journal.pgph.0006119 (PMC12998840; doi:10.1371/journal.pgph.0006119)
Supplement: Attachment — Submitted filename: Response to Reviewers.pdf [file pgph.0006119.s008.pdf]

Response to Reviewers

Dear Editor,

We thank you for your careful assessment of our manuscript and for the constructive feedback provided by both reviewers. We are pleased that the reviewers recognized the relevance, originality, and methodological rigor of our study. We have carefully considered each comment and revised the manuscript accordingly.

Below, we provide a detailed, point-by-point response to all comments raised by the editor and reviewers. All changes are highlighted in yellow in the revised manuscript, and page and line numbers refer to the tracked-changes version.

Editor's Comments

Comment: The reviewers have both noted the strength of your submission and identified reasonable areas for improvement. Please address these to the best of your ability or explain why this was not possible or desirable.

Response: We appreciate the editor's guidance and confirm that all reviewer comments were addressed in full. Revisions were made to improve clarity, coherence, analytic framing, and alignment across sections, particularly in the Introduction, Methods, Results, Discussion, and Conclusion. No recommendations were deemed inappropriate or undesirable to address, and all substantive points were incorporated into the revised manuscript.

Reviewer #1

R1.1 – Introduction structure and factual accuracy

Comment: The introduction would benefit from improved structure, moving from a global overview of TB and TB/HIV to the Brazilian context and the role of civil society. In addition, the opening statement regarding TB as a leading cause of morbidity and mortality since 2023 should be corrected.

Response: We revised the Introduction to improve its logical flow, beginning with a global overview of TB and TB–HIV coinfection, followed by advances and persistent challenges, and then contextualizing the Brazilian setting and the historical role of civil society. We also corrected the statement regarding TB, clarifying that in 2023 TB regained its position as the leading cause of death from a single infectious agent, rather than implying this status began in that year.

Location in manuscript: Introduction, pages 2–4, lines 42–93.

R1.2 – Clear statement of study aim

Comment: The introduction should conclude with a clear statement of the study aims.

Response: We revised the final paragraph of the Introduction to explicitly state the study aim, clearly aligning it with the theoretical framework and the identified gap in the literature.

Location in manuscript: Introduction, page 4, lines 94–100.

R1.3 – Methods: structure and clarity

Comment: The Methods section would benefit from clearer subsection headings and reorganization, including moving the Ethics statement to the end.

Response: We restructured the Methods section using standardized qualitative research headings: Study design; Setting and participants; Data collection; Data analysis; and Ethics statement. The Ethics statement was moved to the end of the Methods section, as suggested.

Location in manuscript: Methods section, pages 4–9, lines 101–236.

R1.4 – Social Representations Theory (SRT)

Comment: Additional explanation is needed regarding the processes of anchoring and objectification, as these are central to the analysis.

Response: We expanded the description of Social Representations Theory, explicitly defining anchoring and objectification and explaining how these processes guided data interpretation. This clarification strengthens the theoretical transparency of the analysis.

Location in manuscript: Methods – Study design, page 5, lines 112–129.

R1.5 – Sampling and recruitment

Comment: Please clarify how civil society organizations and participants were selected and on whose behalf participants were speaking.

Response: We clarified that participant selection was purposive and explicitly described the recruitment process, the types of organizations involved, and the dual positionality of participants, who spoke from both personal lived experience and organizational engagement with TB/HIV care.

Location in manuscript: Methods – Setting and participants, pages 5–6, lines 130–156.

R1.6 – Dates and transcription details

Comment: Use textual dates rather than numeric formats, and explicitly state that interviews were transcribed verbatim in the Data analysis section.

Response: We revised all date references to textual formats (e.g., “between January and July 2025”) and moved the description of verbatim transcription to the Data analysis subsection.

Location in manuscript: Methods – Setting and participants, page 5, line 134; Methods – Data analysis, page 7, lines 197–199.

Reviewer #2

R2.1 – Abstract density

Comment: The abstract is dense and could be more concise, with greater emphasis on what is new.

Response: We revised the abstract to improve readability and conciseness, reducing descriptive density and strengthening the final sentence to more explicitly emphasize the study’s original contribution—namely, framing civil society as co-producers of TB/HIV care.

Location in manuscript: Abstract, page 1, lines 18–36.

R2.2 – Introductory framing of Results categories

Comment: Each Results category should begin with a brief framing statement to orient the reader.

Response: We added short introductory statements at the beginning of each Results category, clarifying how each category emerged from participants’ perceptions and experiences before presenting illustrative excerpts.

Location in manuscript: Results – Category 1, page 10, lines 247–251; Category 2, page 12, lines 302–305; Category 3, page 14, lines 352–355.

R2.3 – Presentation of findings as perceptions

Comment: Findings should consistently be presented as participants’ perceptions and experiences rather than factual claims.

Response: We systematically revised the Results section to ensure consistent use of formulations such as “participants reported,” “participants described,” and “participants perceived.”

Location in manuscript: Results section throughout, pages 9–15.

R2.4 – Verb tense consistency

Comment: Please ensure consistent use of past tense in the Results section.

Response: The Results section was revised to consistently use past tense when reporting participants’ accounts.

Location in manuscript: Results section throughout, pages 9–15.

R2.5 – Overreliance on quotations

Comment: Reduce reliance on long quotations and strengthen analytic framing.

Response: We reduced the length and number of quotations, introduced excerpts more explicitly, and strengthened analytic interpretation in the surrounding text, ensuring quotations illustrate rather than carry the analysis.

Location in manuscript: Results section throughout, pages 9–15.

R2.6 – ART and TPT alignment between Results and Discussion

Comment: ART and TPT are discussed in the Discussion but appear only marginally in the Results.

Response: We clarified in the Results that TPT and ART did not emerge spontaneously as central themes in participants’ narratives. We explicitly interpreted this limited salience through Social Representations Theory, framing TPT as weakly anchored and objectified in shared representations. The Discussion was revised to align with this empirical finding, avoiding overemphasis.

Location in manuscript: Results – Category 1, page 11, lines 285–296; Discussion, page 17, lines 421–437.

R2.7 – Explanation of “territory”

Comment: The term “territory” should be clarified for an international readership.

Response: We added contextual explanation of “territory” as used in the Brazilian public health context, emphasizing its social, political, and service-organization dimensions.

Location in manuscript: Discussion, page 18, lines 452–460.

R2.8 – Threats to civil society organizations

Comment: Clarify whether participants identified threats to NGOs related to funding, sustainability, or longevity.

Response: We expanded Category 3 to explicitly address perceived threats to civil society organizations, including funding instability, lack of institutional recognition, and exclusion from decision-making spaces.

Location in manuscript: Results – Category 3, pages 14–15, lines 389–407.

R2.9 – Redundancy between Discussion and Conclusion

Comment: Reduce repetition and streamline the Conclusion.

Response: We condensed the Conclusion into three focused paragraphs that synthesize the key findings, highlight the study’s novel contribution, and outline implications for policy and practice, avoiding repetition of the Discussion.

Location in manuscript: Conclusion, pages 20–21, lines 511–546.

We believe that these revisions have substantially strengthened the manuscript while preserving its original contribution. We are grateful for the reviewers’ and editor’s insightful feedback and hope that the revised version meets the journal’s expectations.

Sincerely,

Gabriela Tavares Magnabosco,

On behalf of all authors

Corresponding Author
