# Supplementary material for: Civil society perspectives on tuberculosis care for people living with HIV in Brazil: A study informed by Social Representations Theory
Source: PLOS Glob Public Health. 2026 Mar 18;6(3):e0006119. doi: 10.1371/journal.pgph.0006119 (PMC12998840; doi:10.1371/journal.pgph.0006119)
Supplement: Attachment — Submitted filename: Required revisions_PGPH-D-25-03019R1.pdf [file pgph.0006119.s009.pdf]

Required revisions

1. Minimizing Duplication in the Results section

Order effects differed across categories, though themes and interpretations reoccurred. Forcing together repetitive descriptions and limiting redundant quoting will enhance clarity and story flow at no cost to analytical insight.

2. Condense the theoretical definition to the method section

The description of SRT and the important notions for this paper (anchoring and objectification) could be tightened to avoid becoming too esoteric. A more compact definition will preserve the theoretical strictness and be easier to read.

3. Strengthen policy-relevant implications

The implications for policy are general and may be more specific and action-oriented. Explicitly detailing the ways in which civil society can be institutionally incorporated into TB–HIV programmes (e.g., through formal participation in service delivery, prevention, or monitoring) would strengthen the case for the applied public health relevance of this paper.

4. Minor language tightening and polishing

A light editorial edit is advised to condense the text, reduce term repetition, and maintain a consistent scholarly tone throughout.
