# Supplementary material for: Civil society perspectives on tuberculosis care for people living with HIV in Brazil: A study informed by Social Representations Theory
Source: PLOS Glob Public Health. 2026 Mar 18;6(3):e0006119. doi: 10.1371/journal.pgph.0006119 (PMC12998840; doi:10.1371/journal.pgph.0006119)
Supplement: Attachment — Submitted filename: Response_to_Reviewers_R2.pdf [file pgph.0006119.s011.pdf]

RESPONSE TO REVIEWERS

Manuscript ID: PGPH-D-25-03019R1

Title: Civil society perspectives on tuberculosis care for people living with HIV in Brazil: a study informed by Social Representations Theory

Dear Dr. Hoddinott and Reviewers,

We would like to sincerely thank you and the reviewers for your careful and constructive evaluation of our manuscript. We greatly appreciate the insightful suggestions, which have helped us improve the clarity, conceptual framing, and applicability of the manuscript. We have carefully addressed all comments and revised the manuscript accordingly. All changes are highlighted in the tracked-changes version. Below we provide a point-by-point response.

EDITOR COMMENTS

Comment:

The reviewers have raised reasonable suggestions for further editing to strengthen the manuscript.

Response:

We thank the Editor for this assessment. We carefully revised the manuscript to address all reviewer suggestions, including clarifications in the Introduction, improvements in conceptual explanations in the Methods, additional participant information in the Results, refinement of thematic descriptions, and strengthening of policy implications in the Discussion.

REVIEWER #1

We sincerely appreciate the reviewer's positive evaluation and thoughtful suggestions.

Comment 1 — Redundancy between categories

Response:

We carefully revised the Results section to reduce repetition across thematic categories. Overlapping descriptions were consolidated, and redundant quotations were removed or replaced with more analytically illustrative excerpts. These revisions improve narrative flow while preserving interpretive depth.

Comment 2 — Theory section too long

Response:

We condensed the theoretical explanation of Social Representations Theory in the Methods section and streamlined conceptual passages. To enhance clarity for readers unfamiliar with SRT, we added a concrete example illustrating anchoring and objectification processes.

Comment 3 — Policy implications could be more explicit

Response:

We expanded the Discussion to make policy implications more explicit. We now clearly describe how civil society organizations could be formally integrated into TB–HIV programs, including participation in prevention, adherence support, stigma reduction, and monitoring. These additions strengthen the applied relevance of the manuscript.

Comment 4 — Language editing

Response:

The manuscript has undergone careful language editing to improve clarity, reduce wordiness, and eliminate repetitive wording.

REVIEWER #2

We thank the reviewer for the constructive and detailed comments. We have addressed all points as follows.

INTRODUCTION

Comment — Role of civil society needs clearer definition and justification

Response:

We revised the Introduction to:

- provide a clear definition of civil society as used in the manuscript
- describe its role in Brazil's HIV response
- clarify why civil society perspectives constitute an analytical lens for assessing TB–HIV responses

We also strengthened the statement of aims to clarify the meaning of "validating civil society perspectives."

METHODS

Comment — Clarify “It” reference

Response:

We clarified the sentence to explicitly specify whether it refers to Social Representations Theory or to the study design.

Comment — Repetition of anchoring/objectification explanation

Response:

We removed repetitive sentences and replaced them with a concise conceptual explanation plus a concrete illustrative example to enhance reader accessibility.

RESULTS

Comment — Include participant characteristics

Response:

We incorporated participant characteristics into the Results section, including age range, gender distribution, and regional representation. We also corrected the table numbering error and ensured consistency between text and supplementary materials.

Comment — Add concluding reflections for categories

Response:

We added brief interpretive closing paragraphs to Categories 2 and 3 to match the analytical framing already present in Category 1.

DISCUSSION

Comment — TB associated with “dirt”

Response:

We removed this statement to avoid introducing interpretations not directly supported by our empirical data.

Comment — Redundant sentences

Response:

We revised the indicated paragraph to remove repetition and improve concision.

Comment — Transition between TPT and social protection

Response:

We added a linking sentence clarifying the structural relationship between preventive therapy access and broader social protection mechanisms, improving the logical flow of the discussion.

TERMINOLOGY CONSISTENCY

Comment: Check acronyms throughout.

Response:

All acronyms were reviewed and standardized throughout the manuscript.

DATA AVAILABILITY

Comment: Reviewer indicated concern regarding data availability.

Response:

We reviewed and confirmed that the Data Availability statement complies fully with PLOS data policy. It now specifies restrictions due to qualitative data sensitivity and provides a non-author institutional contact point for data access requests.

ADDITIONAL REVISIONS

In addition to the points raised by reviewers, we:

- verified all references for accuracy and consistency
- standardized formatting according to journal requirements
- corrected minor typographical inconsistencies

CONCLUSION

We are grateful for the reviewers' thoughtful feedback and believe the revisions have substantially strengthened the manuscript. We hope the revised version meets the journal's standards and are happy to provide any additional clarification if needed.

Thank you for your consideration.

Sincerely,

Gabriela Tavares Magnabosco, on behalf of all authors
