# Supplementary material for: Civil society perspectives on tuberculosis care for people living with HIV in Brazil: A study informed by Social Representations Theory
Source: PLOS Glob Public Health. 2026 Mar 18;6(3):e0006119. doi: 10.1371/journal.pgph.0006119 (PMC12998840; doi:10.1371/journal.pgph.0006119)
Supplement: Attachment — Submitted filename: Reviewer Report PGPH-D-25-03019_ANG.pdf [file pgph.0006119.s012.pdf]

Reviewer Report – Revision 2

Manuscript: PGPH-D-25-03019
Recommendation: Accept

Overall Assessment

The authors have addressed the concerns raised about earlier versions with diligence and care. The new version is a marked improvement over the old one on all metrics of clarity, structure, and analytical coherence.

The Introduction has now been restructured to include an explicit purpose statement for the study. Specifically, the Methods section provides greater transparency regarding Social Representations Theory and participant recruitment. Results are shorter, and thematic categories are better framed. The Discussion more clearly articulates the policy implications and situates the findings within broader debates on TB–HIV and social protection.

Crucially, the manuscript now presents a clear and well-rooted argument about civil society as co-producers of care within TB–HIV responses. The theoretical framing is carefully couched, and the body of empirical material is consistent with the analytical claims made.

So the remaining issues are purely fine-tuning and purely editorial in nature and do not affect any scientific value, methodological rigor, or interpretive contribution of the study.

Final Recommendation

I believe that the authors have adequately addressed my concerns from previous reviews. The methodology, rigor, conceptual clarity, and relevance of this manuscript make it a strong candidate for publication in PLOS Global Public Health.

Recommendation: **Accept.**
