# Supplementary material for: Civil society perspectives on tuberculosis care for people living with HIV in Brazil: A study informed by Social Representations Theory
Source: PLOS Glob Public Health. 2026 Mar 18;6(3):e0006119. doi: 10.1371/journal.pgph.0006119 (PMC12998840; doi:10.1371/journal.pgph.0006119)
Supplement: Attachment — Submitted filename: PLOS Global Health Review.docx [file pgph.0006119.s007.docx]

**Review of Title: Civil society perspectives on tuberculosis care for people living with HIV in Brazil: a study informed by Social Representations Theory**

**Summary of research and overall impression**

This paper introduces an important topic by examining civil society perspectives on TB and HIV, particularly in a context such as Brazil, where civil society has played a significant role in shaping responses to both epidemics. The study is generally well written and draws on an appropriate methodological framework. However, revisions are needed to strengthen the introduction and results sections in particular, with more minor revisions required in the methods and discussion sections.

**Abstract**

The abstract is clear and well-written, providing a detailed overview of the study.

**Introduction**

The introduction would benefit from revisions to more clearly set up the topic, including a review of its overall structure to improve logic and flow. For example, the authors could begin with a brief global overview of TB and TB/HIV co-infection, including advances in ART and TPT alongside persistent challenges, before moving to a more focused discussion of the role of civil society in HIV responses. This could then be contextualised within Brazil, outlining the HIV/TB situation nationally, the specific role of civil society actors, and why civil society provides an important lens—or “thermometer”—for understanding health and social responses in this setting. The introduction should conclude with a clear statement of the study aims.

In addition, the authors should carefully review specific phrasings for accuracy. For instance, the opening sentence states that TB has been a leading cause of morbidity and mortality since 2023; however, TB has been a leading cause of morbidity and mortality for many years prior to 2023, and this should be corrected.

**Methods**

The methods section is well written, with appropriate attention to relevant methodological detail. The overall approach is methodical and thoughtfully designed. However, a few minor revisions are required to improve clarity and structure.

I suggest revising the section headings to better introduce the different components of the methods: *Study design and setting*; *Sampling and recruitment*; *Data collection*; *Data analysis*; and *Ethics statement* (with the ethics statement moved to the end of the methods section).

Under *Study design and setting*, the authors include a definition of TSR; however, additional detail would be helpful on what is meant by “examining processes of anchoring and objectification,” as this concept is central to the data analysis and interpretation and is referred to later in the manuscript. If available, a figure illustrating this framework could strengthen this section. This subsection should also include a description of the study setting, and the heading should be renamed accordingly.

Please include specific months in the text (e.g., “between January and July 2025”) rather than using date formats such as 29/01/2025. In addition, the description of site selection could be rephrased as “cities were purposively selected based on/considering …”.

Under a separate heading, *Sampling and recruitment*, in addition to the information currently provided under *Participants*, the authors should describe how civil society organisations were selected. If selection was purposive, this should be explicitly stated, along with details on how participants within these organisations were recruited or invited to participate. Provide details to outline who they are speaking on behalf of, as in the results, they seem to be speaking from their own experiences (of care), as well as recounting specific patient stories, and so on.

The *Data collection* section could be moved earlier in the methods (e.g., at line 100), with minor revisions for consistency. This is otherwise a clear and well-written section.

Finally, the *Data analysis* section should explicitly state that interviews were transcribed verbatim, as this information is currently included under *Data collection* but is more appropriately located under *Data analysis*.

**Results**

**General revisions**

For each results category, consider adding a brief introductory statement under the heading to orient the reader and explain how the category was derived from participants’ accounts, before moving into illustrative detail. For example, under Category 1, the authors could begin with a framing sentence such as: *“Participants commonly described perceptions that reflected the social and political erasure of TB …”*

Throughout the results, statements should be revised to ensure that findings are clearly presented as participants’ perceptions and experiences rather than as factual claims (e.g., “participants reported…,” “participants described…,” “participants felt…”). Where relevant, please indicate whether a sentiment was commonly expressed, raised by several participants, or mentioned by only one or a few participants.

Greater contextualisation is needed to integrate the excerpts from the FGDs. At present, there is an overreliance on direct quotations, with insufficient analytic framing. Consider introducing excerpts more explicitly (e.g., “as illustrated in the following FGD excerpt…”), and, where appropriate, supplementing with interview excerpts to strengthen interpretation.

Please review verb tense throughout the results section to ensure consistent use of the past tense rather than the current present tense, as these findings reflect perceptions and experiences reported at a specific point in time (in the past).

Finally, all FGD excerpts should be reviewed for relevance and length, with unnecessary words removed where possible. As noted above, the main text should foreground general patterns and analytic interpretations, with quotations used selectively to add nuance or illustrate key points rather than to carry the analysis.

**Specific revisions**

The first two sentences of the results section could be merged, as the second sentence does not currently stand alone.

The reference to Supplementary File S3 should be revised to S4 (line 145).

In the first paragraph of Category 1, please revise or expand the explanation of the reported lack of knowledge and sense of abandonment by the state, as this theme is currently underdeveloped and not sufficiently substantiated.

ART and TPT are introduced earlier in the manuscript and included in the topic guide, yet they feature only marginally in the results as modes of HIV and TB prevention. Please clarify whether participants discussed how ART as prevention of HIV and TPT as prevention of TB have influenced perceptions of these illnesses, or explicitly note if these themes did not emerge in the data.

The term territory should be clearly explained for an international readership, as its meaning within the Brazilian context may not be widely understood.

Under Category 3, please clarify whether participants identified any threats to civil society organisations or NGOs, particularly in relation to funding, sustainability, or organisational longevity.

**Discussion**

The discussion is generally well written and thorough. However, the links between the study findings and broader patterns of perceptions and experiences need to be articulated more clearly earlier in the manuscript (particularly in the methods and results) and then explicitly revisited in the opening paragraph of the discussion.

As noted in the comments on the results, the discussion expands on TPT and treatment as prevention, yet these themes are only marginally reflected in the results section. Please ensure closer alignment between the results and discussion, either by strengthening the presentation of these findings in the results or by tempering their emphasis in the discussion.

**Conclusion**

There is substantial repetition of similar points across the discussion and conclusion sections. The conclusion would benefit from being streamlined to one to three concise paragraphs that clearly summarise the key messages and contributions of the study, rather than reiterating points already covered in detail in the discussion.
