# Supplementary material for: Civil society perspectives on tuberculosis care for people living with HIV in Brazil: A study informed by Social Representations Theory
Source: PLOS Glob Public Health. 2026 Mar 18;6(3):e0006119. doi: 10.1371/journal.pgph.0006119 (PMC12998840; doi:10.1371/journal.pgph.0006119)
Supplement: Attachment — Submitted filename: PLOS Global Health Review-Round 2.docx [file pgph.0006119.s010.docx]

**Review of Title: Civil society perspectives on tuberculosis care for people living with HIV in** **Brazil: a study informed by Social Representations Theory**

Thank you to the authors for the revised manuscript. The paper has improved, and with some further revisions it could be ready for publication. I outline the remaining issues below, focusing mainly on clarity and positioning.

**Introduction**
Given that the paper centres on civil society, the introduction needs to more clearly introduce and foreground their role in the HIV response. At present, it is still not fully clear how or why civil society is being used as a key lens or benchmark for assessing the response. For an international readership that may be unfamiliar with the Brazilian context (and with civil society’s role in the HIV response more broadly), it would be helpful to include:

- a clear definition of what is meant by “civil society” in this paper,
- an overview of how civil society has been involved in Brazil’s response, and
- a brief explanation of why these perspectives are important for understanding and interpreting the response overall.

Relatedly, the research question and overall aim of the paper would benefit from further clarification, including what is meant by the phrase *“to validate the perspective of civil society.”*

**Methods**
*Study design:*

- In line 72, please clarify whether “It” refers to SRT or to the study itself.
- Lines 77–81 repeat the content of lines 75–77. Rather than restating what anchoring and objectification are, it would be more useful to provide a concrete example to make these concepts more accessible to readers. For instance, an example of “social meaning” and how people might “objectify” this within their everyday social world would help, as these concepts are unlikely to be familiar to many readers.

**Results**

- Please include brief participant details in the results, as currently presented in S1 Table (incorrectly referred to in the text as S2 – line 144). Consider moving this table into the main manuscript rather than the supplement. At minimum, it would be useful to report the overall age range, male/female number/proportion, and if available, years involved in HIV/TB services or civil society (experience).
- The inclusion of TPT in the results is very welcome and clearly illustrates its limited rollout and familiarity.
- The concluding paragraph at the end of Category 1, which highlights TB as being anchored as a forgotten or erased disease, is strong and important. If possible, consider adding similar concluding reflections for Categories 2 and 3.

**Discussion**
The discussion and conclusion have been revised well overall. A few minor points remain:

- Line 294 introduces TB as being associated with “dirt,” which does not appear in the results section. If the authors wish to retain this point, it may be helpful to reference existing literature that has documented this association.
- Lines 287–293 make largely the same point across three sentences and could be tightened.
- Lines 324–329: the paragraph on social protection does not clearly follow from the preceding discussion of TPT. A stronger linking or transition sentence would help the flow here.

**Other points**

- Please check acronyms throughout (e.g., SRT, TB, PLHIV), as their use is currently inconsistent.
